# Rapid RAFT Polymerization of Acrylamide with High Conversion

**DOI:** 10.3390/molecules28062588

**Published:** 2023-03-13

**Authors:** Xuejing Liu, Qiang Sun, Yan Zhang, Yujun Feng, Xin Su

**Affiliations:** 1 CNOOC Key Laboratory of Offshore Drilling Fluids and Cementing, China Oilfield Services Limited, Sanhe 065201, China; 2 Polymer Research Institute, State Key Laboratory of Polymer Materials Engineering, Sichuan University, Chengdu 610065, China

**Keywords:** RAFT, acrylamide, water-soluble polymer, controllable living radical polymerization

## Abstract

Rapid RAFT polymerization can significantly improve production efficiency of PAM with designed molecular structure. This study shows that ideal Reversible Addition–Fragmentation Chain Transfer (RAFT) polymerization of acrylamide is achieved in dimethyl sulfoxide (DMSO) solution at 70 °C. The key to success is the appropriate choice of both a suitable RAFT chain transfer agent (CTA) and initiating species. It is illustrated that dodecyl trithiodimethyl propionic acid (DMPA) is a suitable trithiocarbonate RAFT CTA and is synthesized more easily than other CTAs. Compared to other RAFT processes of polymers, the reaction system shortens reaction time, enhances conversion, and bears all the characteristics of a controlled radical polymerization. The calculation result shows that high concentrations can reduce high conversions, accelerate the reaction rate, and widen molecular weight distributions slightly. This work proposes an excellent approach for rapid synthesis of PAMs with a restricted molecular weight distribution.

## 1. Introduction

Polymers that dissolve or swell in water to generate solutions or dispersions are called water-soluble polymers [1]. The hydrophilicity of water-soluble polymers is attributable to the presence of hydrophilic groups inside their molecules. The most prevalent hydrophilic groups include carboxyl, hydroxyl, amide, amine, and ether, among others. Not only do these groups render the polymer hydrophilic, but they also expand its variety of uses. Not only may the strength and number of hydrophilic groups be tailored to specific needs, but their active functional groups can also be reacted to produce compounds with novel functional groups. The variety of water-soluble polymers [2] and their distinctive qualities have led to a rise in their use in industry, agriculture, medicine, and other disciplines.

Polyacrylamide (PAM) is the collective term for homopolymers and copolymers of acrylamide (AM) and its derivatives [1]. It is one of the most extensively used water-soluble polymers. AM, as a monomer, is carcinogenic, but its polymer is non-toxic; hence, PAM is widely utilized in a variety of sectors. PAM is the most abundant and versatile water-soluble polymer, often prepared by conventional free radical polymerization [1], which results in water-soluble polymers with generally high molecular weights, but with uncontrollable structures and wide molecular weight distributions of the polymerization products. However, it is difficult to remove the reaction heat and the kinetics of jelly-like polymerization cannot be adequately controlled, resulting in PAM with a broad molecular weight dispersion. A portion of the low-molecular-weight PAMs in this product performs poorly as a flocculant and thickener, hence limiting the use of homogeneous aqueous solution polymerization. With the requirement for the refinement of water-soluble polymers, PAM with controllable structure and molecular weight has vast potential applications. It is difficult to achieve this goal using conventional free radical polymerization, but the emergence and development of controllable/reactive free radical polymerization has provided new possibilities.

Controlled radical polymerization (CRP) techniques [3], including Atom Transfer Radical Polymerization (ATRP) [4,5,6,7,8], Nitroxide–Mediated Polymerization (NMP) [9], and Reversible Addition–Fragmentation Chain Transfer Polymerization (RAFT) [10,11,12,13], have been the focus of intense research in recent years due to their versatility and potential commercial applications. CRP polymerization can provide PAM with predetermined molecular weights, limited molecular weight dispersion, and intricate macromolecular structures. Grassl et al. [14] effectively homopolymerized AM utilizing the NMP approach with a regulated polymerization process and moderate reaction conditions, but the initiator was costly and difficult to manufacture on a large scale. Jewrajka et al. [15] used ATRP method in a mixture of glycerol and water solvent (1:1, *v*/*v*) to achieve controlled polymerization of AM. However, the low monomer conversion, long reaction time, and tendency of amide monomers to poison the metal catalysts in the ATRP solution hinder the achievement of controlled/reactive radical polymerization.

RAFT polymerization proves itself to be an extremely versatile CRP technique. Rizzardo et al. [16,17], from Australia, initially introduced the notion of RAFT polymerization in 1998. Corpart et al. [18] presented a similar idea, Macromolecular Design through the Exchange of Xanthates, for the production of polymers via sulfonate ester transfer exchange. In nature, both polymerization processes are reversible fracture-transfer radical polymerization. Since then, there has been an explosion of RAFT polymerization research globally. In light of the variety of applications and the high demand for water-soluble polymers, the synthesis of water-soluble polymers with a narrow molecular weight distribution, controllable structure, and low molecular weight has become the direction of development for controlled/reactive radical polymerization. However, in the synthesis of water-soluble polymers, the NMP method has few applicable monomers and harsh polymerization conditions, limiting its applicability. ATRP is typically performed in a mixture of water and organic solvents or in organic solvents and is inapplicable to the polymerization of amide monomers [19,20,21].

In the study of RAFT polymerization, the creation of water-soluble polymers by this approach has caught the interest of academic and industrial researchers; however, the synthesis of water-soluble polymers by RAFT polymerization has only been reported in industrial production on rare occasions. The following discussion will thus focus on the application of RAFT polymerization to the manufacture of water-soluble polymers. It demonstrates applicability to the controlled polymerization of a broad variety of monomers under a broad range of circumstances to produce materials with specified molecular weights, restricted molecular weight distributions, and sophisticated architectures [17,18,19,20,21,22,23,24,25,26,27,28,29]. McCormick et al. [30] determined that RAFT appears to be the most promising approach for polymerizing acrylamide (AM) in aqueous settings. They used sodium dithiobenzenesulfonate 4–cyanopentanoate as a chain transfer agent (CTA) in a buffer solution of acetic acid/sodium acetate (pH = 5) with VA–086 as an initiator to successfully synthesize PAM with controllable molecular weight and molecular weight distribution; however, the reaction time was lengthy and the conversion was only 28% at 24 h of reaction. The polymerization of AM monomers by conventional CRP methods has been shown to be troublesome [31], but acrylamide monomers are readily polymerized by RAFT [32]. It is reported that polymerizations of AM by RAFT require a considerable amount of time, meaning conversions are quite low [30,31,32,33]. 

In this work, the CRP of AM in DMSO was performed using RAFT polymerization, and the kinetic characteristics of the polymerization reaction as well as the effects of monomer concentration and chain transfer agent concentration on the polymerization process are discussed.

## 2. Results and Discussion

### 2.1. Synthesis of DMPA

**Scheme 1:**
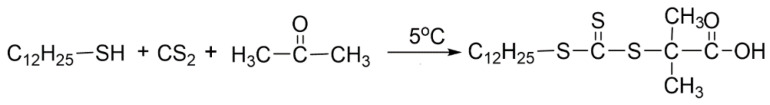
The synthesis procedure of chain transfer agent, DMPA.

**Figure 1:**
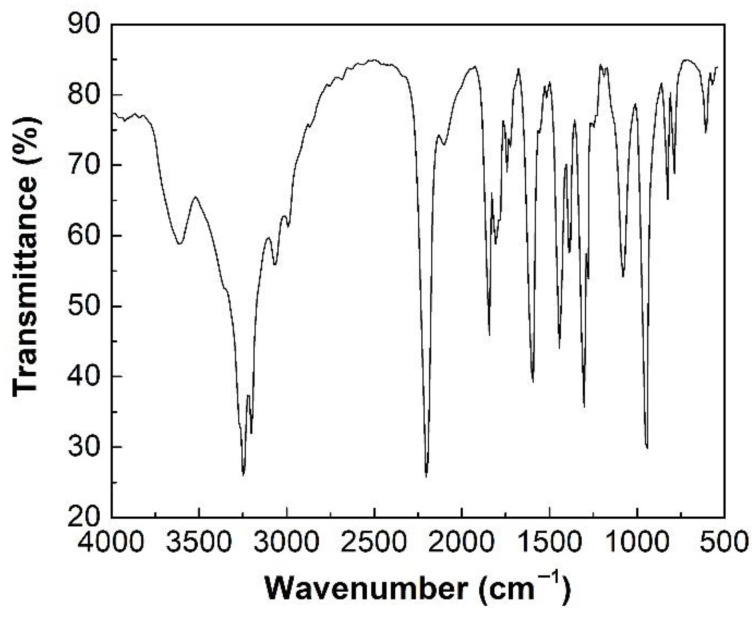
The FTIR spectrum of chain transfer agent, DMPA.

**Figure 2:**
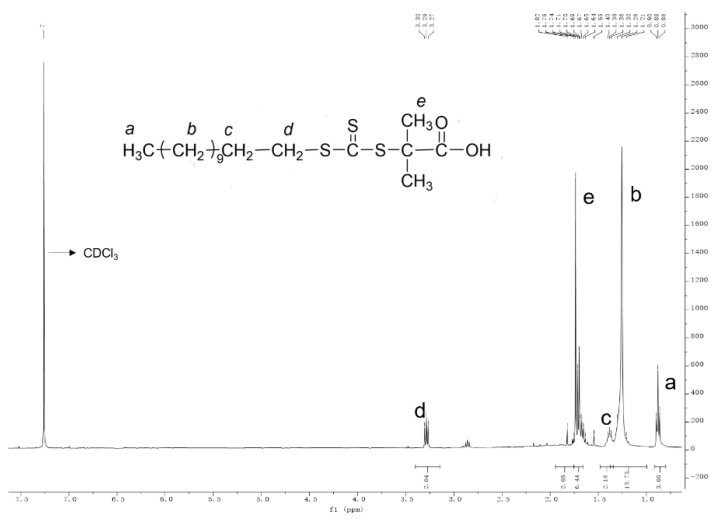
The ^1^H NMR spectrum of chain transfer agent, DMPA.

For RAFT polymerization of AM, a suitable RAFT CTA is the primary consideration. Dodecyl trithiodimethyl propionic acid (DMPA) has been shown to be a good RAFT CTA that is more readily produced (Scheme 1) than other CTA. Figure 1 presents the FTIR spectrum of DMPA. There is a strong –OH stretch in 3449 cm^−1^, a strong C=O stretch in 1715.3 cm^−1^ and a strong C=S in 1068.3 cm^−1^; all the signals indicate the existence of DMPA. Figure 2 demonstrates the ^1^H NMR spectrum of DMPA: 0.90 ppm (t, 3H), 1.25–1.40 ppm (m, 20H), 1.72 ppm (s, 6H), 3.27 ppm (t, 2H), showing that the desired DMPA has been achieved.

### 2.2. RAFT Polymerization of AM

Dimethyl sulfoxide (DMSO) was chosen as solvent for RAFT. After a number of fruitless efforts employing a mixture of 2–propanol and water (1/4, *v*/*v*) as solvent, it was determined that the polymerization process could not occur in this mixture because 2–propanol acted as a transfer agent.

**Figure 3:**
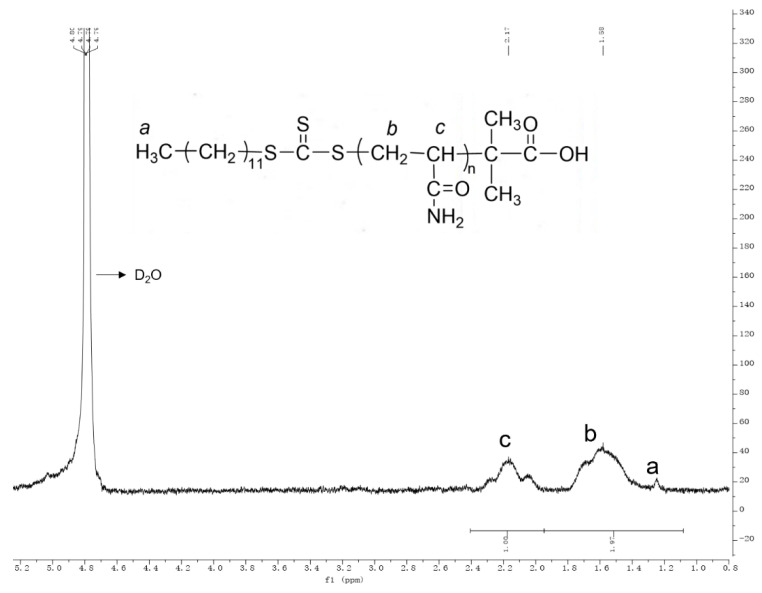
The ^1^H NMR spectrum of PAM prepared via RAFT process in D_2_O.

To demonstrate the effectiveness of RAFT, the molecular structure of the PAM produced after polymerization was studied via ^1^H NMR. Figure 3 depicts the ^1^H NMR spectrum of PAM in D_2_O, which contains three major absorption peaks: the absorption peaks at 1.6 ppm and 2.0 ppm are the chemical shifts of protons in –CH and –CH_2_ on the main chain of PAM, respectively, and the integrated area ratio of these two absorption peaks is close to 1:2, which coincides with the corresponding number of H atoms in the molecular structure of PAM. The chemical shift of the –CH_2_–proton should be between 1.3 and 1.7 ppm, but it is obscured by the chemical shift of the proton in the main chain; however, the tiny absorption peak at 0.9 ppm is obviously the absorption peak of the proton in the end methyl group, which indicates the existence of a C_12_H_25_–tail chain at the end of the polymer, and thus it can be concluded that DMPA has successfully played the role of CTA and the RAFT polymerization process has been performed.

Table 1 depicts the molecular weight and dispersity (Đ = M_w_/M_n_) of PAM at various times during RAFT. The actual molecular weight of PAM is quite close to its theoretical molecular weight; the molecular weight distributions of the samples at various times are narrow (Đ < 1.3). This is consistent with features of CRP.

**Figure 4:**
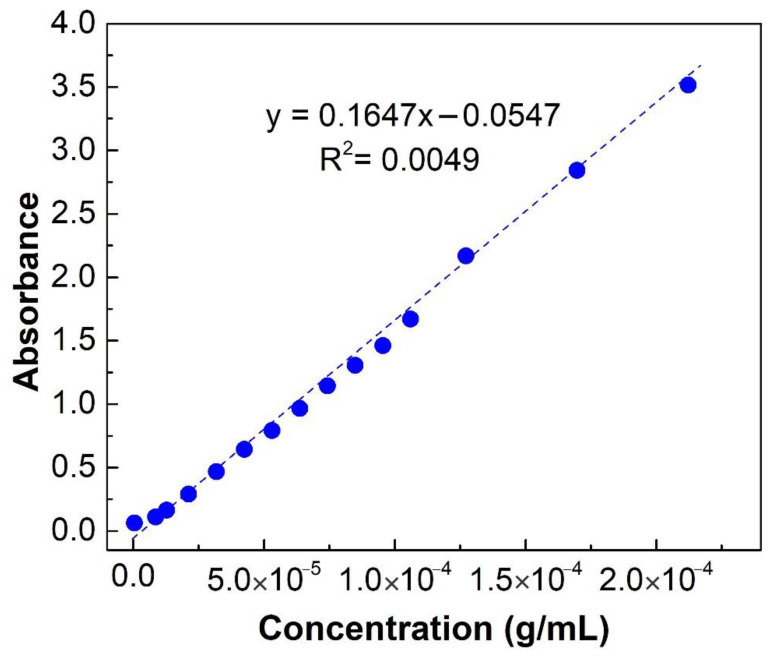
Absorbance plotted as function of concentration for AM in ethanol.

The reaction rate of RAFT polymerization must be established based on the monomer conversion. Conversion was determined by ultraviolet (UV) spectrophotometer. The fluctuation of the absorbance of the ethanol solution of AM with concentration is seen in Figure 4. In accordance with the Beer–Lambert law, it has been determined that AM has a good linear relationship in the low concentration range, therefore the AM content in the reaction system at different times may be computed using this standard curve in Figure 4.

**Figure 5:**
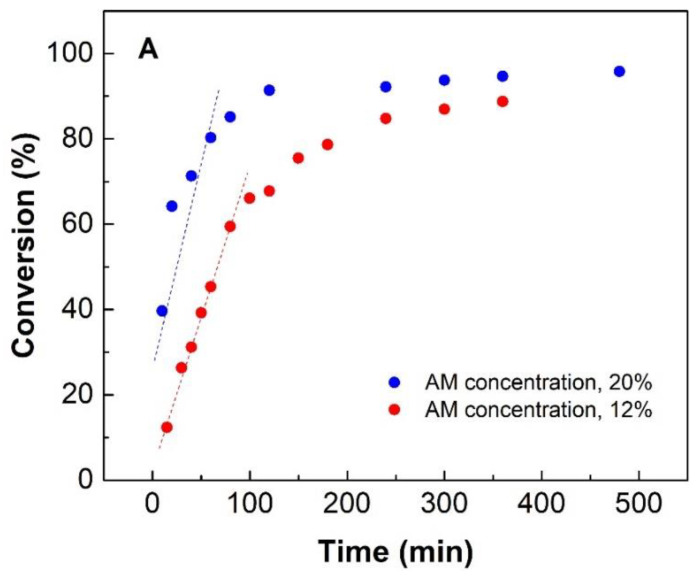

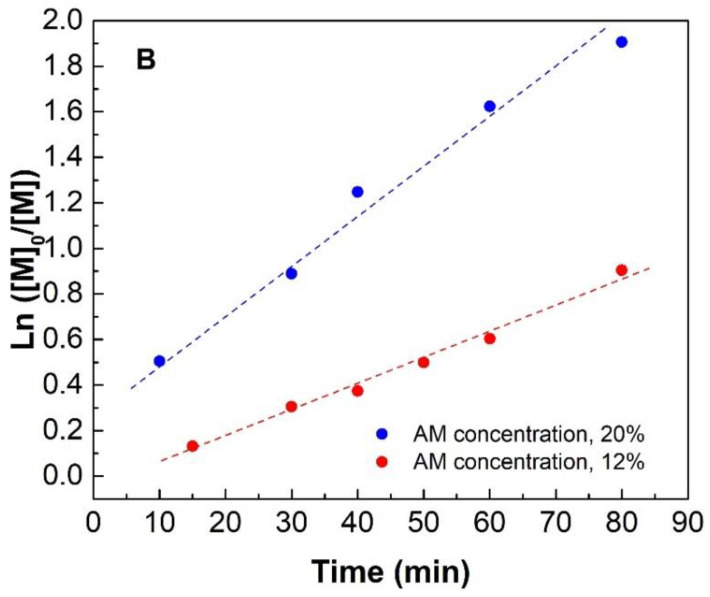
The kinetics plot of RAFT of AM with different concentrations (12% and 20%), including plot of conversion vs. time (**A**) and plot of ln([M]_0_/[M]) vs. time (**B**). Polymerization conditions: [M]_0_/[CTA]_0_/[AIBN]_0_, 200/1/0.1; temperature, 70 °C.

The kinetics of the polymerization reaction is one of the great indicators of whether the RAFT polymerization process matches CRP. The link between number average molecular weight and monomer conversion is seen in Figure 5. The conversion of monomer to polymer grows linearly with reaction time, and the number average molecular weight of the polymer also increases linearly with conversion. Due to the high reaction temperature, the primary radicals were produced virtually instantly, and the radicals increased gradually in the presence of chain transfer agents, while the reversible-break transfer process successfully regulated the incidence of double-group termination. Consequently, the above kinetic results show that RAFT polymerization of AM may be accomplished utilizing DMPA as the chain transfer.

### 2.3. Influence of Monomer Concentration

Rarely has the influence of monomer concentration on controlled/reactive polymerization been studied in the extant literature. In this work, the [M]_0_/[CTA]_0_/[AIBN]_0_ ratio was maintained at 200/1/0.1, and monomer concentrations of 12% and 20% were chosen to examine the impact of monomer concentration on the polymerization process (Figure 5).

High monomer concentration was observed to result in a rapid polymerization rate. At t = 120 min, for instance, the monomer conversion was 70% when [M]_0_ = 12% and 90% when [M]_0_ = 20%. This is mostly due to the increased monomer concentration, which enhances the likelihood of free radical collision with monomers and facilitates chain formation, hence accelerating the conversion. In addition, the conversion inflection point arises earlier under conditions of high monomer concentration. McCormick et al. [13] also investigated RAFT polymerization AM; they employed a dithio chain transfer agent with a monomer concentration of 14.2%. However, the reaction time was lengthy and the monomer conversion was low; the monomer conversion at 24 h was only 28%. In addition, when the reaction time was extended, the molecular weight distribution grew dramatically. The dispersion of molecular weight expanded as the reaction time increased. Our research utilized DMPA was as a chain transfer agent to polymerize AM in DMSO. At a monomer concentration of [M]_0_ = 12% and a reaction period of 120 min, the conversion was as high as 70%, the reaction time was dramatically reduced, and the conversion was significantly raised. This may be related to the increased chain transfer activity of DMPA and the increased reversible-break transfer rate. In addition, Figure 5B demonstrates that the polymerization process at various doses is consistent with the controlled/reactive radical polymerization features.

It was also discovered that as the monomer concentration is elevated, the Đ of molecular weight expands. For instance, the Đ of the same sample at 120 min with [M]_0_ = 20% was 1.47, whereas the Đ of the same sample with [M]_0_ = 12% was 1.23. When the monomer concentration is high, there is a larger possibility of collision between the free radical and the monomer, which results in a quicker polymerization rate and increases the likelihood of double-radical termination, which leads to a broader molecular weight dispersion. However, Đ values below 1.5 are still compatible with features of CRP. From the preceding analysis, it is clear that AM concentration is a significant factor influencing the RAFT polymerization: a high monomer concentration results in a rapid polymerization reaction, but the Đ of molecular weight widens, making it difficult to obtain monodispersed PAM of high quality.

### 2.4. Influence of CTA

**Figure 6:**
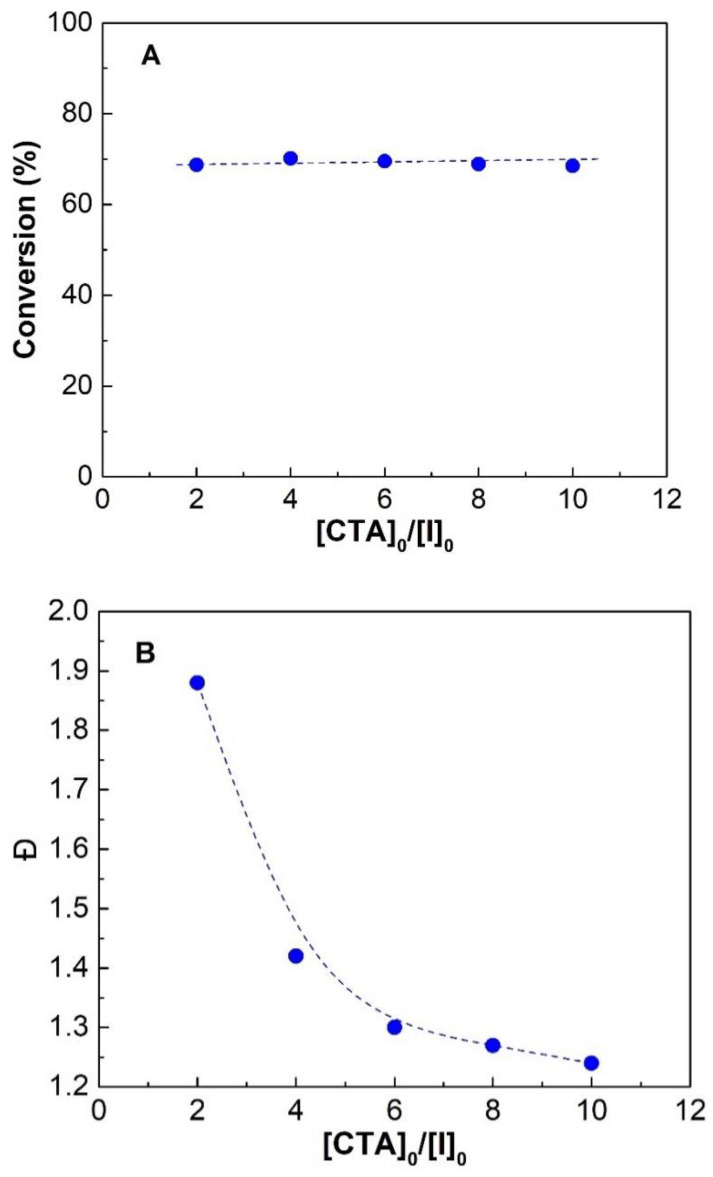
Conversion vs. CTA (**A**) and Đ vs. CTA (**B**). M_0_/I = 200/0.1, 70 °C, 200 r min^–1^, T = 70 °C.

To accomplish CRP, the amount of RAFT chain transfer agent is crucial. The RAFT polymerization of AM was discovered to be possible using DMPA. Figure 6 demonstrates that when the monomer concentration and initiator concentration are held constant and the chain transfer agent concentration is altered, the conversion of AM remains nearly the same, but the Đ of PAM changes significantly: the high ratio of chain transfer agent to initiator monomer substance decreases the Đ value of obtained PAM. This may be explained by polymerization activity of AM and the RAFT polymerization characteristics: AM is a monomer with significant polymerization activity, and once the reversible-break transfer occurs, polymerization ceases. When the polymerization process is established, the amount of CTA added cannot reduce its reaction rate; therefore, the amount of CTA does not affect the monomer conversion, whereas during polymerization, the initiation dose is constant, i.e., the number of primary radicals generated does not change. The added chain transfer agent causes a successful RAFT process and the polymerization process minimizes the likelihood of double group termination, regulating the Đ of molecular weight.

### 2.5. Influence of Temperature

**Figure 7:**
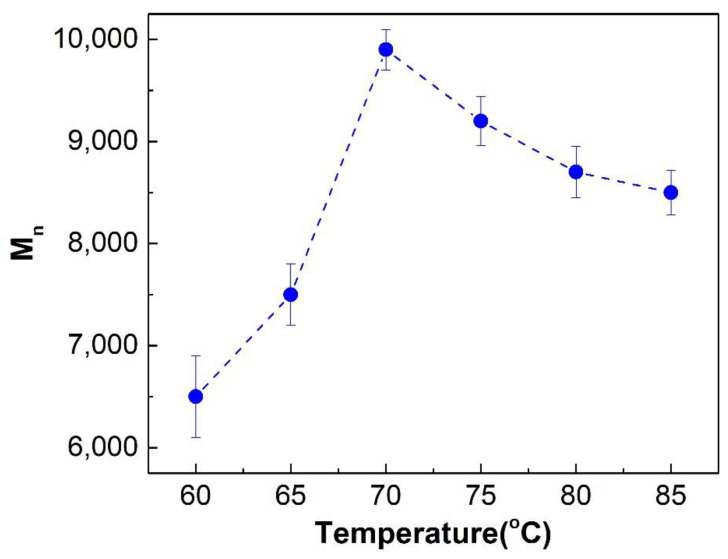
The effect of reaction temperature on molecular weight of PAM. The plot of M_n_ vs. polymerization time. Polymerization conditions: [M]_0_, 12%; [M]_0_/[CTA]_0_/[AIBN]_0_, 200/1/0.1; temperature range, 60 °C to 85 °C.

The impact of temperature on the molecular weight of synthesized PAM was studied under the identical circumstances of initiation dose, CTA and initiator molar ratio, and reaction duration, as illustrated in Figure 7.

The influence of reaction temperature on the molecular weight of produced PAM is depicted in Figure 7. The graph illustrates that when temperature increases, the molecular weight of PAM tends to first grow and subsequently drop. This is because the free radicals generated by the decomposition of the initiator (AIBN) increase as the temperature rises, thereby accelerating the chain growth rate, and the surplus free radicals can rapidly react with the C=S group on CTA to form the dormant intermediate, which can quickly establish a chemical equilibrium with the active species to maintain a stable concentration of free radicals in the polymerization system. The dormant intermediates can rapidly establish chemical equilibrium with the reactive species, maintain a steady radical concentration in the polymerization system, limit the likelihood of double radical termination, and thus efficiently regulate the pace of polymer chain formation. As the temperature climbs from 70 °C to 85 °C, the molecular weight of PAM decreases. The reason is that the temperature continues to rise and the polymerization process becomes progressively more difficult to initiate. The decomposition rate of the initiator is too rapid as the temperature continues to rise, resulting in a high concentration of free radicals in the polymerization system. The excess free radicals are unable to react completely with the CTA to form dormant intermediates. The research above demonstrates that when the reaction temperature is 70 °C, the produced PAM has a larger molecular weight.

### 2.6. Influence of Initiator

The initiator is a molecule with a structure of weak bonds that, when exposed to heat or radiation energy, breaks into free radicals, therefore initiating the polymerization of monomers. The initiator primarily plays two roles: the first is to decompose to produce free radicals, which are the active center of the polymerization reaction; the greater the number of free radicals, the greater the number of active centers of the polymerization reaction, and the lower the molecular weight of the polymer. The second is to accelerate the speed of the polymerization reaction, increasing the conversion rate of the monomer, thereby making the reaction more complete.

**Figure 8:**
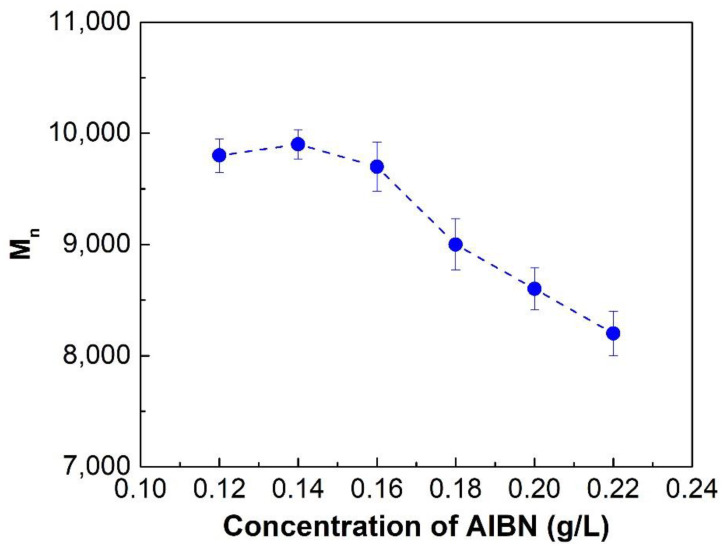
The effect of AIBN concentration on molecular weight of PAM. The plot of M_n_ vs. polymerization time. Polymerization conditions: [M]_0_, 12%; [M]_0_/[CTA]_0_, 200/1; range of [AIBN], 0.12 to 0.22 g/L; temperature, 70 °C.

The reversible addition break reaction between chain growth radicals and chain transfer agents is the cornerstone of RAFT polymerization. Since the radicals in the reaction are provided by the decomposition of foreign initiators, the selection and dosage of initiators are the determining factors for controlling the rate of the polymerization reaction; therefore, the dosage of the initiator has some effect on the molecular weight of the synthesized products. In this experiment, AIBN was selected as the initiator, and the effect of initiator dosage on the molecular weight of the synthesized PAM was investigated under the same conditions of reaction temperature, molar ratio of chain transfer agent and initiator, and reaction time; the experimental results are depicted in Figure 8.

The impact of the initiator dose on the molecular weight of produced PAM is seen in Figure 8. The graph illustrates that the molecular weight of PAM decreases when the amount of initiator is excessive or insufficient. When the amount of initiator is below 0.14 g/L, the initial concentration of AIBN is lower, the induction period is longer, the monomer cannot be completely initiated during the reaction, the concentration of growth radical is too low (resulting in a slower polymerization rate), and the degree of polymerization reaction proceeds at a slower rate, leading to an incomplete polymerization reaction and a lower molecular weight. With an increase in initiator dose, the concentration of primary radicals and growth radicals created by the chain transfer of primary radicals rises, therefore accelerating the polymerization reaction rate, increasing the monomer conversion rate, and increasing the molecular weight of PAM. When the initiation dosage surpasses 0.14 g/L, the concentration of free radicals in the system is excessive, and the likelihood of double radical termination increases, leading to a reduction in molecular weight. Based on the above investigation, it is evident that when the initiator concentration is 0.14 g/L, the produced PAM has a greater molecular weight.

### 2.7. Molar Ratio of CTA and Initiator 

Under identical circumstances of reaction temperature, initiator dose, and reaction time, the effect of the molar ratio of chain transfer agent to initiator on the molecular weight of the synthesized PAM was examined.

**Figure 9:**
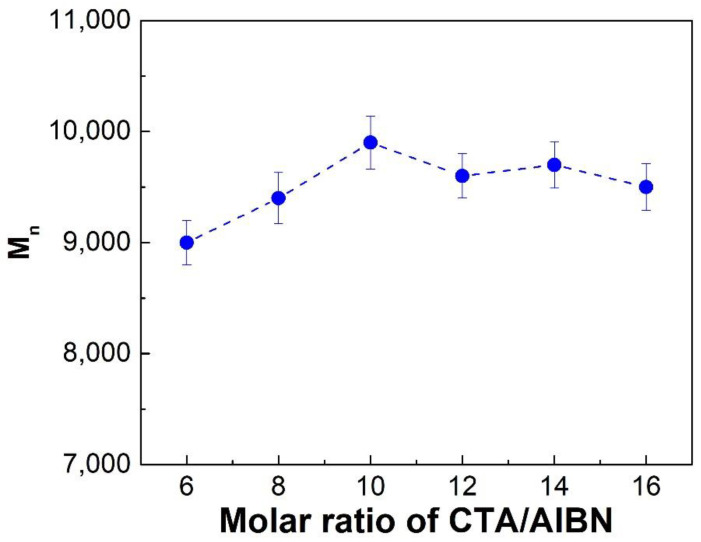
The effect of molar ratio of CTA and initiator on molecular weight of PAM. The plot of M_n_ vs. polymerization time. Polymerization conditions: [M]_0_, 12%; range of [CTA]_0_/[AIBN]_0_, 6 to 12; temperature range, 60 °C to 85 °C.

Figure 9 illustrates the effect of the CTA to AIBN molar ratio on the molecular weight of produced PAM. The plot demonstrates that as the molar ratio increased, the molecular weight of PAM exhibited a growing and subsequently declining trend. When the molar ratio of chain transfer agent CTA to AIBN is lower, the concentration of chain transfer agent in the polymerization system is relatively low, which is not conducive to the equilibrium shift during the chain growth phase, resulting in a lower concentration of dormant intermediates generated in the system. In addition, the increased concentration of free radicals in the system raises the likelihood of coupling termination of the reaction and decreases its controllability, resulting in a lower molecular weight.

As the molar ratio of CTA to AIBN increased, the concentration of initial chain transfer agent in the system increased, and more initial chain transfer agents participated in the chain transfer reaction, which ensured that the chain transfer agents in the system could react with the growth radicals in time, keeping the concentration of growth radicals in the system essentially unchanged so that the polymerization reaction was more precisely controlled and the molecular weight increased. However, when the molar ratio of CTA to AIBN surpassed 10, the concentration of RAFT reagent was too high and an increased number of chain transfers occurred during the polymerization process, resulting in a decrease in molecular weight. When the molar ratio of CTA to AIBN is 10, the synthesis of PAM is enhanced, and the molecular weight of the PAM produced is greater.

By accumulating and comparing the data of the existing RAFT for preparing PAM (Table 2), it was discovered that the PAM preparation described in this study achieves rapid RAFT reaction and high AM conversion. Within 120 min, conversion of 92% may be obtained. All other documented RAFT procedures need a minimum of three hours. Moreover, the RAFT of PAM homopolymer is seldom documented, and of the few studies that do exist, the RAFT in DMSO for PAM homopolymer described in this study belongs to a quicker process.

## 3. Materials and Methods

### 3.1. Materials

Acrylamide (AM) was bought from Changjiu Agri–Scientific Co., Ltd. (Nanchang, China) recrystallized from ethanol for two times. 2,2–Azobis(isobutyronitrile) (AIBN) as the initiator was supplied from Tianjin Kemiou Chemicals (Tianjin, China), recrystallized from methanol, and the purity was more than 99%. The solvents, including DMSO, methanol, ethanol, and acetone, were all purchased from Guanghua Chemicals (Guangdong, China) and used directly without any further purification. The water used in this work was distilled three times. The nitrogen had a purity of 99.99%.

### 3.2. Synthesis of DMPA

The chain transfer agent, DMPA, was synthesized according to the literature following the reported procedure [38]. Dodecanethiol (20.19 g), acetone (46.4 g), and tetrabutylammonium bromide (1.33 g) were added into a four-necked flask; the mixture was cooled to 5 °C in an ice-water bath. The solution containing CS_2_ (7.7 g) and acetone (11.6 g) was slowly dropped into the flask. After stirring for 20 min, the solution in the flask gradually turned bright yellow. The reaction continued for 20 min, and the solution became clear from turbidity; chloroform (17.8 g) and NaOH aqueous solution (50%, 40.0 g) were then added. After 24 h of reaction, distilled water (150 mL) and concentrated hydrochloric acid (37%, 25 mL) were added, and a large number of rice-like yellow particles appear above the solution. The yellow solid was washed in 300 mL isopropanol, and the filtrate was concentrated into a viscous paste, which was recrystallized in n–hexane three times. After drying, a yellow crystal was obtained at 16.18 g, which was the target product of dodecyl trithiodimethyl propionic acid (DMPA).

Crystalline solid; IR (KBr): 3449.8 cm^−1^, 1715.3 cm^−1^, 1068.3 cm^−1^. ^1^H NMR: 0.90 (t, 3H), 1.25–1.40(m, 20H), 1.72 (s, 6H), 3.27 (t, 2H).

### 3.3. RAFT Polymerization of Acrylamide

The typical polymerization procedure was as follows: After dissolving the AM, DMPA, and DMSO in a flask, N_2_ was passed into the solution for 30 min to remove O_2_, while the solution was heated to 70 °C. The designed amount of AIBN solution was then added, and the polymerization was processed under N_2_ for 2 h, then cooled in cold water, and a portion of the reaction solution was taken with a separatory funnel. After extraction, the precipitate was precipitated with acetone and dried under vacuum at 50 °C for 24 h. Polymerization kinetics and molecular weights were determined by GPC from the polymerization solution at predetermined time intervals, at which points the reacting solution was immediately quenched by immersion in ethanol. Conversions at different time were calculated by the absorbance at 242 nm between start to certain reaction time.

### 3.4. Characterization

Number and weight average molecular weights, M_n_ and M_w_, respectively, and molecular weight distributions (Đ = M_w_/M_n_) were determined by Gel Permeation Chromatography (GPC) using a Waters 515 pump, Ohpak KB–803 column and Waters 2410 refractive-index (RI) detector. The equipment was calibrated with polyethyleneoxide standards, and a phosphate buffer (pH = 6.5) at 0.10 mol/L was used as the eluent with 0.80 mL/min flow rate. Conversions of monomers (C) were determined by U–2010 spectrophotometer (Hitachi, Tokyo, Japan). ^1^H NMR (300 MHz) spectra were recorded on a Bruker Avance 300 spectrometer. A Nicolet MX–1E IR spectrophotometer was used to record the IR spectra in the scanning range of 4000 to 400 cm^–1^. Conversion was determined by ultraviolet (UV) spectrophotometer. The solution of AM was put into different 50 mL volumetric flasks, then diluted into different concentraioninto with 80% ethanol solution. Their absorbance was measured at 242 nm, and the absorbance–concentration curve was obtained as Figure 1. Aliquots were removed after 0, 20, 40, 80, 100, and 120 min, which were diluted by ethanol/water (4:1, *v*/*v*) prior to analysis. Conversions were determined comparing the signal of the absorbs at 242 nm.

### 3.5. Determination of Molecular Weight and Molecular Weight Distribution

Additionally, it was necessary to compare the measured molecular weight and theoretical one (M_n,th_) to determine the success of RAFT. The measured and theoretical molecular weight and conversion data at different times are listed in Table 1. M_n,th_ of PAM was calculated from the conversion of monomer, the initial molar concentration ratio of the monomer to CTA, the molecular weight of AM (71.08), and the molecular weight of DMPA (348.56). M_n,th_ at different reaction times can be calculated by the number of units of monomer converted into polymer, as shown by Equation (1):

(1) $$M_{n,th}=71.08\times conversion\times {\left[M\right]}_{0}/{\left[CTA\right]}_{0}+348.56$$

## 4. Conclusions

According to the findings of this research, a decently regulated RAFT of acrylamide may be accomplished in DMSO at 70 °C by combining a conventional initiator (AIBN) with DMPA as the CTA. According to our findings, a high concentration not only decreases the high conversion and quick velocity, but also results in a somewhat wider molecular weight dispersion. It is evident that the procedure that was reported in the current work offers a substantial gain in the capability to achieve high conversion in a short amount of time, therefore preparing a block of PAM to be included in a future investigation.

This study examines several factors for RAFT polymerization. The chain transfer agent facilitates RAFT, and polymerization reduces double group termination, controlling molecular weight. Excess free radicals cannot completely react with CTA to create inactive intermediates. RAFT polymerization at 70 °C has a higher molecular weight, according to studies. When the initiator concentration is 0.14 g/L, it is clear that the generated PAM has a higher molecular weight. When the molar ratio of CTA to AIBN is 10, the synthesis of PAM is accelerated and the molecular weight of the PAM synthesized is higher.

## Figures and Tables

**Table 1:** Kinetic and molecular weight data for the RAFT polymerization of acrylamide (([M]_0_/= 12%, [CTA]_0_/[AIBN]_0_ = 10:1)).

Polymerization Time (min)	Conversion (%)	M_n_(theory) ^a^ (g/mol)	M_n_(exp) ^b^ (g/mol)	M_w_(exp) ^b^ (g/mol)	Đ ^b^
40	30.2	4640	4550	5690	1.25
60	44.0	6610	5790	7140	1.23
80	54.7	8180	6430	8070	1.25
100	62.3	9210	7920	10,030	1.27
120	70.1	10,300	8700	10,800	1.24

^a^ Data were obtained from conversion; ^b^ Data were determined by GPC.

**Table 2:** Comparison of the results of different RAFT processes for PAM preparation.

No.	1	2	3	4	5	6
Type of PAM	Homo Polymer	Homo Polymer	Homo Polymer	Homo Polymer	Block Polymer	Block Polymer
Nature of CTA	Dodecyl trithiodimethyl propionic acid	2-(2-thiobenzoylsulfonylpropionylamino)ethanesulfonate	2-(2-Thiobenzoylsulfonylpropionylamino)ethanesulfonate	S,S″-Bis (α, α′-dimethyl-α’’-acetic acid) trithiocarbonate	S,S″-Bis (α, α′-dimethyl-α″-acetic acid) trithiocarbonate	3-benzyl-trithiocarbonyl propionic acid
Concentration of AM (mol·L^−1^)	2.8	2.0	2.0	0.7	7.1	0.63
Molar ration AM/CTA/I	200/1/0.1	920/1.15/1	4000/5/1	3000/10/1	7075/8.25/1	359.7/2/1
Reaction Temperature (°C)	85	70	70	45	70	50
Polymerization time (min)	120	1440	1200	450	560	240
Conversion (%)	92	28	75	78	99	95
M_n_ (g/mol)	10,920	28,900	80,000	14,100	13,890	48,600
Ref.	This work	[33]	[34]	[35]	[36]	[37]

## Data Availability

The data presented in this study are available on request from the corresponding author.

## Sample Availability

Samples of the compounds are not available from the authors.
